# Small RNA sequencing of circulating small extracellular vesicles microRNAs in patients with amyotrophic lateral sclerosis

**DOI:** 10.1038/s41598-023-32717-y

**Published:** 2023-04-04

**Authors:** Jin-Ah Kim, Canaria Park, Jung-Joon Sung, Do-Jin Seo, Seok-Jin Choi, Yoon-Ho Hong

**Affiliations:** 1 Department of Neurology, Seoul National University Hospital, Seoul, Republic of Korea; 2 Department of Translational Medicine, Seoul National University College of Medicine, Seoul, Republic of Korea; 3 Genomic Medicine Institute, Medical Research Center, Seoul National University, Seoul, Republic of Korea; 4 Department of Neurobiology, Harvard Medical School, Boston, MA USA; 5 Department of Neurology, Chung-Ang University College of Medicine, Seoul, Republic of Korea; 6 Department of Neurology, Neuroscience Research Institute, Medical Research Council, Seoul National University College of Medicine, Seoul National University Seoul Metropolitan Government Boramae Medical Center, Seoul, Republic of Korea; 7 Department of Neurology, Seoul National University Seoul Metropolitan Government Boramae Medical Center, 20 Boramaero-5-Gil, Dongjak-Gu Seoul, 07061 Republic of Korea

**Keywords:** Cell biology, Neuroscience, Systems biology

## Abstract

Dysregulation of microRNAs (miRNA) in small extracellular vesicles (sEV) such as exosomes have been implicated in the pathogenesis of amyotrophic lateral sclerosis (ALS). Although circulating cell-free miRNA have been extensively investigated in ALS, sEV-derived miRNAs have not been systemically explored yet. Here, we performed small RNA sequencing analysis of serum sEV and identified 5 differentially expressed miRNA in a discovery cohort of 12 patients and 11 age- and sex-matched healthy controls (fold change > 2, p < 0.05). Two of them (up- and down-regulation of miR-23c and miR192-5p, respectively) were confirmed in a separate validation cohort (18 patients and 15 healthy controls) by droplet digital PCR. Bioinformatic analysis revealed that these two miRNAs interact with distinct sets of target genes and involve biological processes relevant to the pathomechanism of ALS. Our results suggest that circulating sEV from ALS patients have distinct miRNA profiles which may be potentially useful as a biomarker of the disease.

## Introduction

Amyotrophic lateral sclerosis (ALS) is a fatal and heterogeneous neurodegenerative disease with no known cure^1^. It causes a relentlessly fast-progressing motor neuron loss which leads to generalized skeletal muscle wasting, spasticity and ultimately death due to respiratory failure within 2–5 years after symptom onset^2^. Most cases are sporadic, while up to 10% of patients have a family history^3^. The pathogenesis is incompletely understood with more than 40 genes identified as being causative or associated with ALS^4^.

Diagnosis of ALS is a clinically driven exclusion process which often results in considerable diagnostic delay in part due to a wide variability of clinical phenotypes and the lack of reliable biomarkers^5^. Therefore, effective biomarkers that can reflect heterogeneity and pathophysiology of ALS may help to expedite diagnosis and better design therapeutic trials^6^.

MicroRNAs (miRNA) are endogenous small non-coding RNAs (18–23 nucleotides long) which regulate gene expression at post-transcriptional level^7^. Emerging evidence suggests that dysregulation of miRNA is implicated in the pathomechanism of ALS^8^. A number of biomarker studies have been undertaken to identify circulating cell-free miRNAs in ALS^9^. Notably, however, very few miRNA overlap across different studies, which may reflect biological heterogeneity of the disease, small size of study cohort and methodological differences in sample preparation and miRNA profiling^8^.

Small extracellular vesicles (sEV) such as exosomes and microvesicles are nano-sized (40–100 nm) lipid membrane vesicles released by various cell types which contain bioactive molecules such as nucleic acids and proteins^10^. A growing body of evidence has implicated sEV as an important platform for intercellular communication in various physiological and pathological processes^11^. Studies have shown that sEV can transfer ALS-associated proteins such as SOD1 and TDP-43 and may induce the prion-like propagation of protein misfolding and aggregates^12,13^. Exosome-mediated transfer of miRNA from neuron has been demonstrated to regulate glutamate transporter-1 expression in astrocyte (and thereby extracellular glutamate levels) and phenotypic alterations in microglia^14,15^. Given the stability in various body fluids and protection of enclosed RNA from enzymatic degradation, sEV may be a more reliable source of biomarker than free-floating circulating miRNAs^16^.

Here, we present the results of small RNA sequencing analysis of circulating sEV miRNA from ALS patients and age- and sex-matched healthy controls. Differentially expressed miRNAs were further evaluated using droplet digital PCR (ddPCR) in an independent cohort. To assess the pathophysiological relevance of validated miRNAs, bioinformatic analysis was performed for the miRNA-target interaction and biological pathways enriched in the target genes.

## Results

### Clinical characteristics

A total of 30 patients with ALS (12 discovery set and 18 validation set) and 26 controls (11 discovery set and 15 validation set) were enrolled. The participants’ demographic and clinical features were summarized in Table 1. There were no significant differences in age and sex between the ALS and control groups in both the discovery and validation sets. Although patients in discovery set showed slightly longer disease duration and lower ALSFRS-R score than validation set, all patients showed a typical clinical course of ALS during at least 18 months of follow-up. We only included cases of definite, probable, or probable laboratory-supported ALS, excluding possible ALS. Additional demographic information was provided in Supplementary Table 1.

**Table 1:** Clinical features of study participants.

	Discovery set		Validation set	
	ALS (n = 12)	Control (n = 11)	ALS (n = 18)	Control (n = 15)
Sex (M:F)	5:7	5:6	11:7	9:6
Age (years)	59 (37–76)	58 (52–75)	60 (52–76)	67 (53–77)
Disease duration (months)	17 (9–31)	–	5.5 (3–10)	–
Onset region				
Bulbar	6 (50%)	–	8 (44.4%)	–
Limb	6 (50%)	–	10 (55.5%)	–
ALSFRS-R*	30.5 (18–46)	–	39 (19–45)	–
Level of diagnostic certainty				
Definite	5		12	
Probable	4		4	
Probable laboratory-supported	3		2	

Data are expressed as median value (min–max).

*ALS* amyotrophic lateral sclerosis, *M* male, *F* female, *ALSFRS-R* ALS functional rating scale-revised.

### Characterization of serum-derived small EV enriched fractions

**Figure 1:**
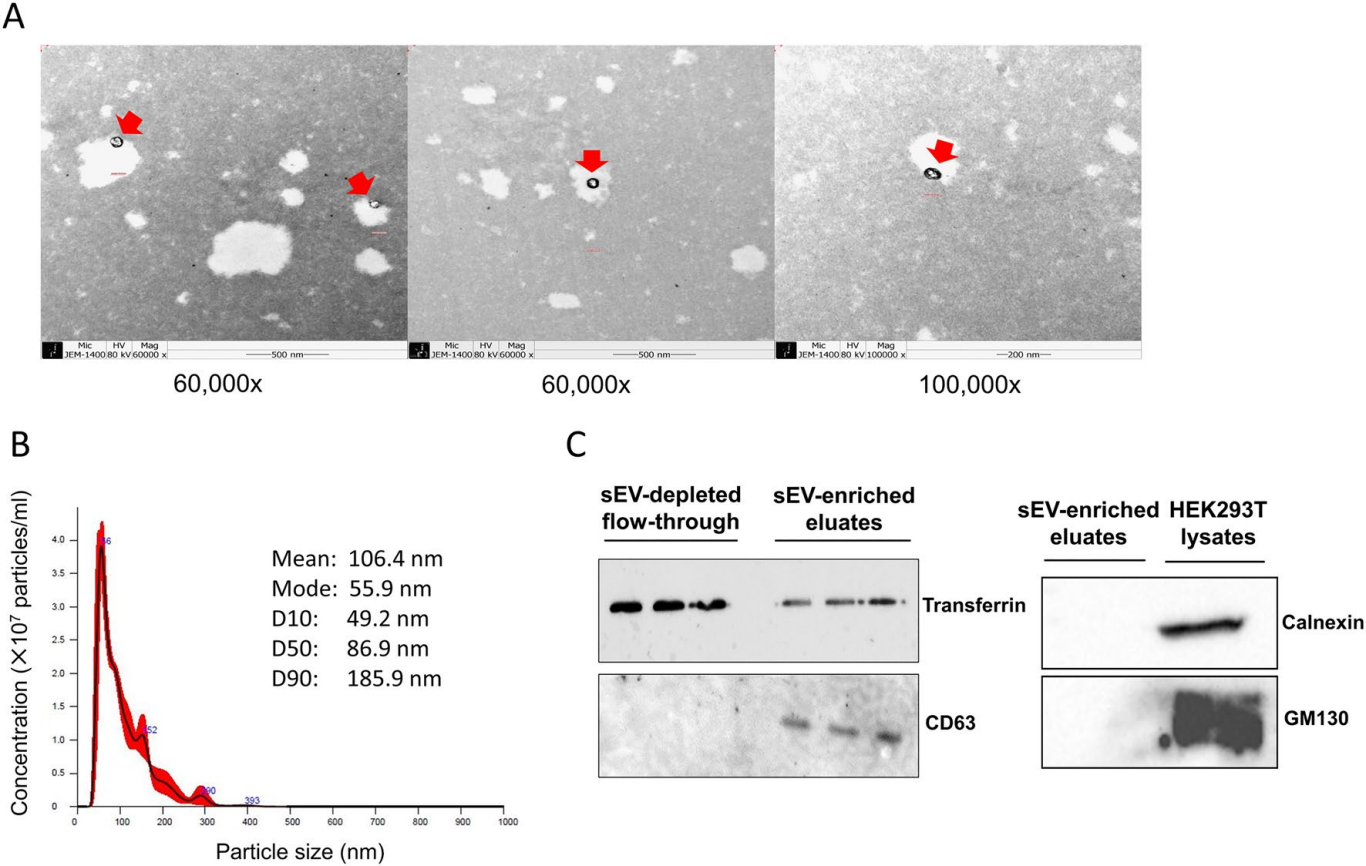
Characterization of serum-derived small extracellular vesicles (sEV). (**A**) Representative transmission electron microscopy (TEM) images of sEV isolated by exoEasy kit. Scale bars are 500 nm on the left and middle images (magnification × 60,000) and 200 nm on the right image (magnification × 100,000). Spherical membranous particles with an expected size of sEV are indicated with red arrows. (**B**) Representative particles size distribution profile of nanoparticle tracking analysis (NTA). The calculated size distribution is depicted as a mean (black line) with standard error (red shaded area) along with descriptive summary of particles size. The mean concentration of particles (± standard error) was 2.73 × 10^9^ (5.71 × 107) particles/ml. Data were analyzed using NanoSight NTA software v3.2 (https://www.malvernpanalytical.com/en/support/product-support/software/nanosight-nta-software-update-v3-2). (**C**) The expression of CD63 (exosome marker) and transferrin (negative marker) in sEV-enriched eluate and sEV-depleted flow-through was determined by western blotting. The expression of calnexin and GM130 (both negative markers) was also examined in sEV eluate and cell lysate (HEK293T). Full scans of all uncropped images are provided in Supplementary Fig. 1.

The sEV enriched fractions were extracted from the serum by using a commercial kit (exoEasy Maxi Kit, QIAGEN). The morphology and size distribution of sEV were analyzed by transmission electron microscopy (TEM) and nanoparticle tracking analysis (NTA), which showed circular-shaped vesicles with sizes ranging from 50 to 300 nm (mean 138 nm, mode 87 nm) (Fig. 1A,B). The mean concentration of particles (± standard error) was 2.73 × 10^9^ (5.71 × 10^7^) particles/ml. In western blotting assays, CD63 (exosome marker) was detected in the sEV enriched fractions, but not in the sEV-depleted fractions. Conversely, transferrin, a negative marker for exosome revealed much lower intensity in the sEV-enriched eluates than those in the sEV-depleted fractions. Calnexin and GM130 (negative markers) were absent both in sEV-enriched eluates and cell lysates (Fig. 1C).

### Small RNA composition of serum EV enriched fractions

We first tested 24 samples (12 ALS patients and 12 healthy controls) in the discovery set by small RNA sequencing. No less than 14.88M reads were produced for each sample, of which 20.3–62.5% included the adapter sequence with the read length of 18 bp or more after trim (trimmed read) and 0.1–5.3% did not include adapter (non-adapter read) (Supplementary Table 2).

Excluding rRNA, which accounted for 38–74% of the total processed reads, the median proportion of known miRNAs was 1.52% of the processed reads mapped to the category of small RNA (Supplementary Fig. 2). We excluded one control sample (C7) for the subsequent analysis because of the low number of mapped reads to miRbase precursor (< 10,000).

### Differential expression of sEV miRNAs between ALS patients and healthy controls

To identify differentially expressed sEV miRNAs (fold change > 2, p < 0.05), we analyzed a total of 266 known miRNAs detected in at least 50% of all samples for both patient and control groups. Since there is no established gold standard method for normalization and statistical testing of small RNA sequencing data, we applied three different methods commonly used for differential gene expression analysis, i.e., DESeq2 (RLE and negative binomial Wald test), edgeR (TMM and exact test) and quantile normalization with independent *t* test.

**Figure 2:**
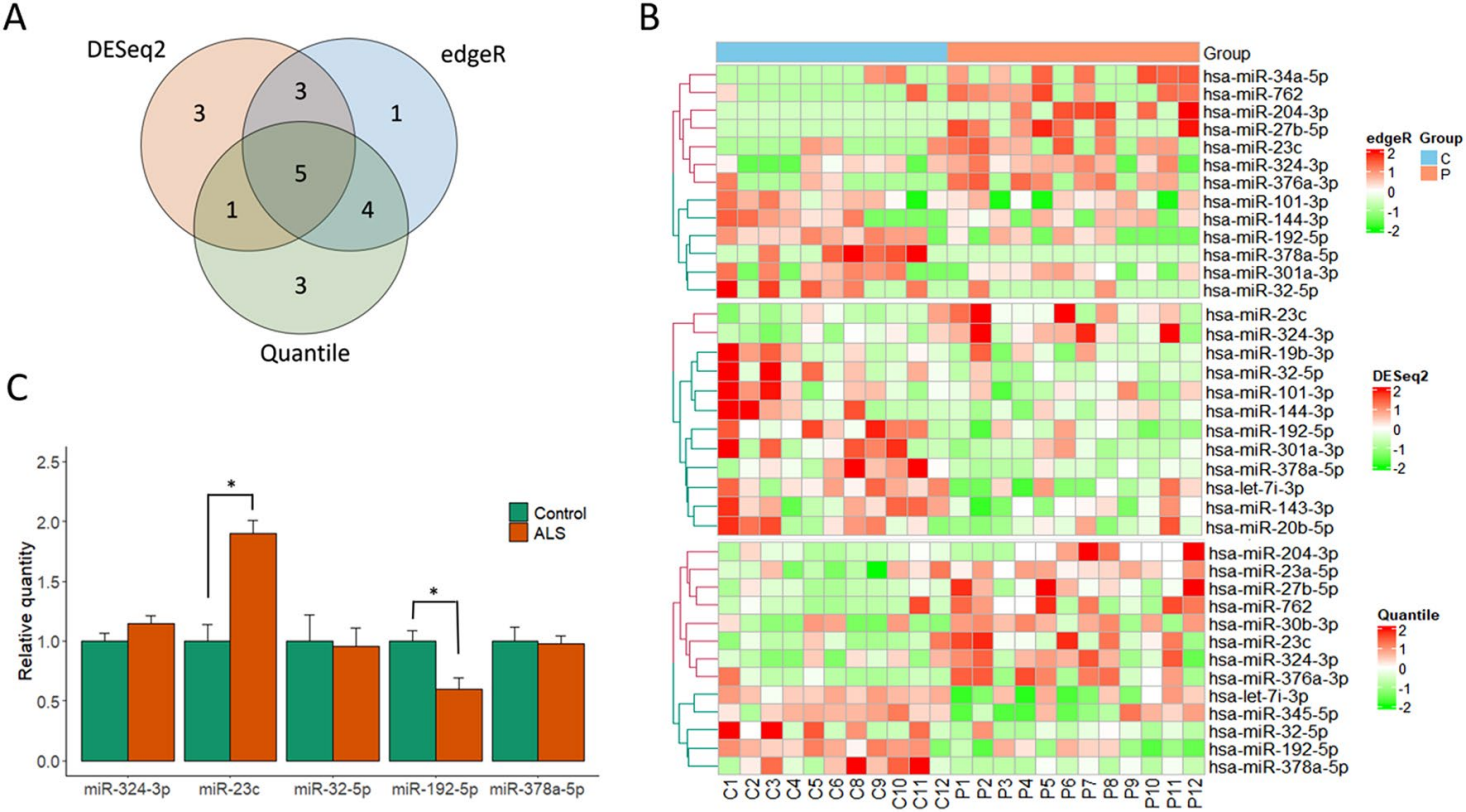
Differential expression of sEV miRNAs and the results of droplet digital PCR validation. Venn diagram (**A**) and heatmaps (**B**) showing the number and differential expression of miRNAs identified in a discovery set by each method (DESeq2, edgeR, and quantile normalization with independent *t* test). Expression values were normalized across all samples (columns: P, ALS patients; C, healthy controls) by Z-score, and miRNA clustering (row) was applied. (**C**) Quantification of levels of five miRNAs (commonly identified in the three analyses) by droplet digital PCR in a validation set. The amplified droplets were analyzed using Quantasoft software v1.7.4 (https://www.bio-rad.com/webroot/web/pdf/lsr/literature/10047467.pdf). Each bar indicates the relative mean quantity of each miRNA (normalized to miR-191-5p as an endogenous reference) compared to the average value in the healthy control. Error bars and asterisk (*) represent standard error of the mean and statistically significant difference (p < 0.05, Wilcoxon rank sum test).

When the overall results are combined, 20 miRNAs were found differentially expressed in ALS patients compared to controls (12 in DESeq2, 13 in edgeR, 13 in quantile normalization) (Fig. 2A,B). To validate the differential expression, we selected 5 miRNAs commonly found in all three methods: 2 up-regulated miRNAs (miR-23c, miR-324-3p) and 3 down-regulated miRNAs (miR-192-5p, miR-32-5p, miR-378a-5p). Next, we measured the expression of 5 miRNAs by droplet digital PCR (ddPCR) in a separate cohort of 18 subjects and 15 controls, which confirmed the up- and down-regulation of miR-23c and miR192-5p, respectively (Fig. 2C).

We evaluated the diagnostic performance of the two dysregulation miRNAs and the correlations with clinical parameters. Binary logistic regression models were built with miR-23c, miR192-5p and both. The results of ROC curve analysis were provided in Supplementary Fig. 3 and Supplementary Table 3. Our results suggested that a greater diagnostic performance could be achieved by combination of both miRNAs in a logistic model. The estimated Spearman’s correlation coefficients between the normalized level of miRNA and baseline clinical parameters were provided along with multiple correlation plots in Supplementary Fig. 4 and Supplementary Table 4. We could not find any significant clinical correlations including onset age, disease duration and revised ALSFRS total score.

### Target gene prediction and functional enrichment analysis

To investigate pathophysiologic relevance of the 2 differentially expressed and validated miRNAs, we performed functional enrichment analysis of their target genes. By using the multiMIR package in R, we systemically screened eight predicted and three validated miRNA-target interaction databases and identified 545 and 135 genes targeted by miR-23c and miR-192-5p, respectively.

**Figure 3:**
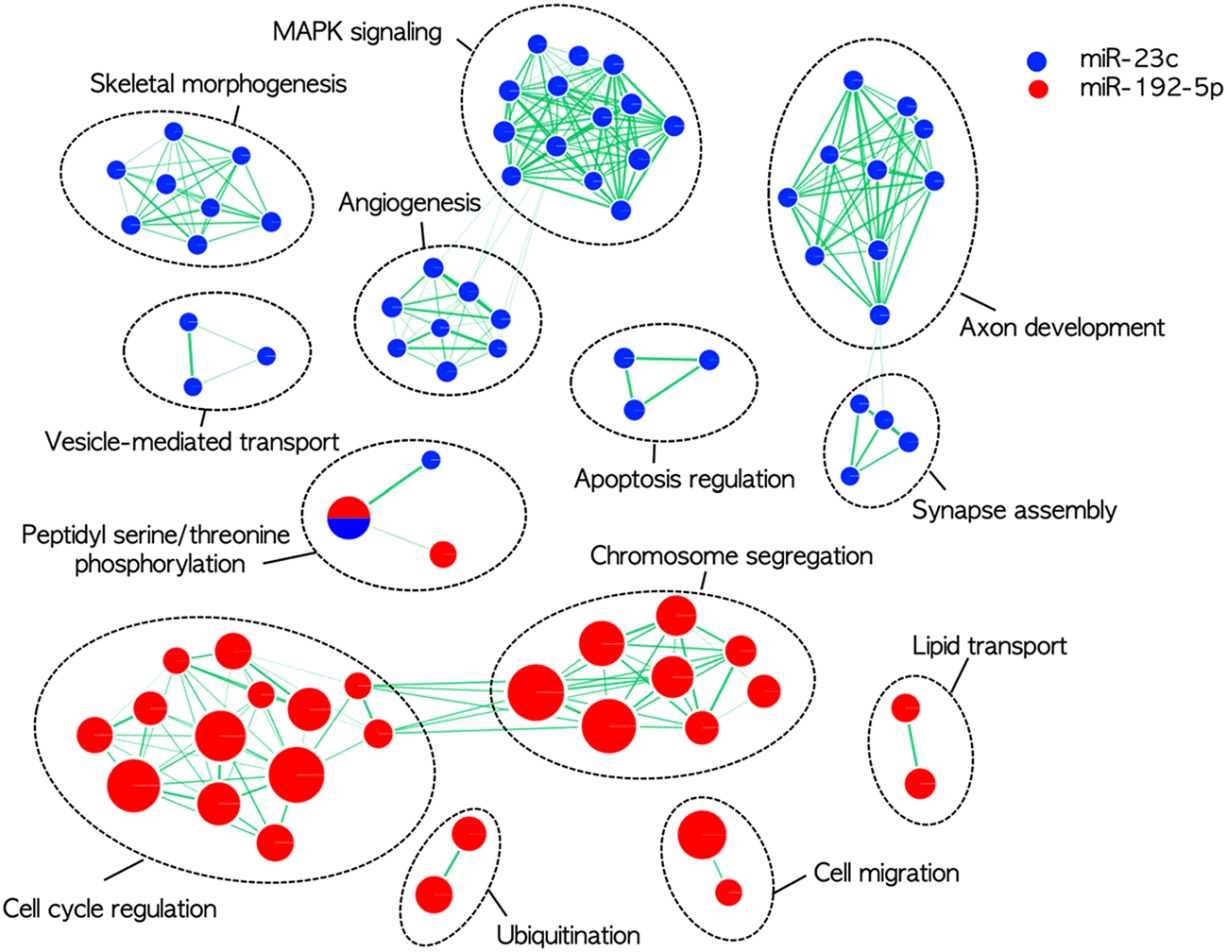
Enrichment map illustrating significantly over-represented pathways (Gene Ontology biological process) for the genes targeted by miR-23c (blue) and miR-192-5p (red), respectively. Enrichment results were mapped as a network of gene sets (nodes) which are grouped together as a subnetwork by their shared genes. Node size is proportional to the total number of genes within each gene set. Edge represents the gene overlap between gene sets, and its thickness is proportional to size of the overlap.

Functional enrichment analysis using Gene ontology biological process revealed distinct lists of over-represented pathways for the two miRNA target gene sets (FDR < 0.05); For miR-23c, MAPK signaling, axon development, synapse assembly, apoptosis, and vesicle-mediated transport. For miR-192-5p, cell cycle regulation, chromosome segregation, ubiquitination, cell migration and lipid transport (Fig. 3).

## Discussion

In this study, we isolated sEV from serum by membrane-based affinity binding using a commercial kit (exoEasy), and demonstrated that they are mostly comprised of exosomes with regards to physicochemical properties including the morphology, size and marker proteins. Through an unbiased systematic analysis of miRNAs using small RNA sequencing, we identified 5 miRNAs differentially expressed in ALS patients compared to healthy controls. These miRNAs were further examined using ddPCR in an independent cohort, which confirmed the up- and down-regulation of miR-23c and miR-192-5p, respectively, in ALS patients. Bioinformatic analysis revealed that these two miRNAs may interact with distinct sets of target genes which involve biological processes potentially relevant to the pathomechanism of ALS.

Although there are many biomarker studies on circulating miRNAs in ALS patients, exosomal miRNAs have been investigated in only a few studies with no overlapped results across studies. Likewise, the two validated miRNAs in present study, miR-192-5p and miR-23c, have not been identified in previous studies^17–22^. The inconsistency might be due to a combination of technical and biological factors including different methods for blood sample preparation (serum vs. plasma), EV isolation (precipitation, immuno- or peptide affinity, or a combination of both), miRNA profiling (microarray, RNA sequencing, NanoString), bioinformatic analysis, and biological variability along with disease heterogeneity. We added a summary table to compare methods used in studies of EV miRNA biomarker in ALS (Supplementary Table 5). While there are a variety of different methods for isolating EV, all methods have both pros and cons and the choice would depend on the specific needs and experimental conditions. The optimal method of sEV isolation from human blood is not determined yet. Although ultracentrifugation allows for greater control over the size selection of EV, it is labor-intensive and often results in low yield^23,24^. Requiring large sample volumes, ultracentrifugation may not be feasible in most clinical studies where small sample volumes are available^24^. Thus far, there have been 6 published studies investigating circulating EV miRNAs in ALS, but none of them used ultracentrifugation for isolating sEV from serum or plasma.

Interestingly, we found that there was little overlap for the target genes and their implicated functional pathways of the two validated miRNAs, suggesting that miR-23c and miR-192-5p may be involved in distinct sets of biological processes. As for miR-23c, the most enriched biological pathways were closely related with mitogen-activated protein kinase (MAPK) signaling which is implicated in a wide variety of cellular processes including differentiation, proliferation, and apoptosis^25^. In ALS, the activation of p38 MAPK has been proposed to mediate the toxic effects of SOD1 and FUS through glial activation, increase of nitric oxide production, and hyperphosphorylation of neurofilaments^26–28^. Inhibition of p38 alpha was demonstrated to revert the axonal transport deficits in SOD1 G93A mouse model^29^.

The cell cycle regulation and chromosomal segregation are the most enriched biological pathways for the miR-192-5p target genes, which is in line with numerous studies demonstrating significant up-regulation of miR-192-5p in malignant tumors^30,31^. Of note, emerging evidence has implicated the members of cyclin-dependent kinase (Cdk) family in ALS^32,33^. Deregulation of Cdk5 activity is associated with the hyperphosphorylation of tau and neurofilament proteins in SOD1 (G37R) mice^34^. Cdk4, a regulator of the G1-S checkpoint, is also upregulated and associates with the abnormal phosphorylation of retinoblastoma protein in SOD1 (G37R) mice^32^. Inhibition of Cdk5 activity by overexpression of the Cdk5 inhibitory peptide in motor neurons significantly improves the pathology and extends survival in SOD1 (G37R) mice^35^. MiR-192-5p interacts with more than 20 genes belonging to Cdk family including Cdk4 and Cdk5^31^, suggesting that down-regulated miR-192-5p may be related to the alteration of cell cycle regulation and subsequent neuronal death.

Our study has some limitations. First, the sEV isolation method used in this study (exoEasy) may not be optimal particularly in terms of purity. Although it has been reported to isolate highly pure RNA with high specificity for vesicular over non-vesicular RNA^36^ and we determined the quality and quantity of eluted EVs by a range of different methods (TEM, NTA, and western blotting), comparison of RNA profiles using NGS in both EV-enriched and depleted fractions may be required for verification. Second, the exact cellular sources of serum sEV cannot be identified. Recently, two studies have investigated neuron-derived EV miRNA signatures in plasma by enriching EV through immunoaffinity purification using neural adhesion molecule (L1CAM)^18,20^. However, there was no overlap between dysregulated EV miRNAs from these two studies, which might be due to technical variations in the enrichment process. Third, we could not find any significant correlations between dysregulated miRNAs and clinical parameters including disease severity (as measured by the revised ALSFRS) and duration (Supplementary Fig. 4 and Supplementary Table 4). This might be related to disease heterogeneity and/or insufficient statistical power of the study. Since EV miRNA biomarker research in ALS is currently challenged by a variety of technical and biological factors affecting the outcomes, collecting evidence from studies of different methods and cohorts is paramount. Although our results need to be confirmed in larger cohorts, we believe that our small RNA-seq study can provide an unbiased and comprehensive sEV miRNA profile and help in the design of future studies.

In conclusion, our results suggest that circulating sEV in ALS patients have distinct miRNA profiles which may be potentially useful as a biomarker of the disease. Further works in larger samples are warranted to confirm the diagnostic potential of miR-23c and miR-192-5p in sEV from ALS patients.

## Methods

### Participants

Serum samples were obtained from ALS patients and age- and sex-matched healthy controls at the Seoul National University Hospital (Seoul, Republic of Korea). The discovery cohort consisted of 12 ALS patients and 11 healthy controls, and the validation cohort included 18 ALS patients and 15 healthy controls. Diagnosis of ALS was made according to the revised El Escorial criteria^37^. Written informed consent was obtained from each participant. This study was approved by the local institutional review board of Seoul National University Hospital (IRB No. 1706-016-856), and was conducted in accordance with the Declaration of Helsinki.

### Isolation of small extracellular vesicles (sEV)

Blood was drawn from the antecubital vein (BD Vacutainer SST tube, SKU #367986), left to clot at room temperature, and centrifuged at 2700 rpm for 10 min at 4 °C within 2 h. The supernatant was aliquoted and stored at − 80 °C (the Seoul National University Hospital Human Biobank). Small extracellular vesicles (sEV) were isolated by using exoEasy Maxi Kit (QIAGEN GmbH, Hilden, Germany) according to the manufacturer’s instructions. Briefly, 4 ml of pre-filtered serum (0.8 μm syringe filter, MilliporeSigma, USA) was mixed 1:1 with buffer XBP and added to the exoEasy membrane affinity spin column. After centrifugation (500*g* for 1 min), 10 ml buffer XWP was added to the column and centrifuged at 5000*g* for 5 min. The flow-through was used as the sEV-depleted fraction for western blot analysis. Then, 400 μl buffer XE was added to the membrane, incubated for 1 min and centrifuged at 500*g* for 5 min to collect the sEV eluate. We have submitted all relevant data of our experiments to the EV-TRACK knowledgebase (EV-TRACK ID: EV220323)^38^.

### Nanoparticle tracking analysis

The concentration and size distribution of sEV were determined by nanoparticle tracking analysis on NanoSight LM10 configured with 405 nm laser and CCD camera (Malvern Panalytical) according to the manufacturer’s instructions. 400 μl of sEV eluate was injected to the sample chamber for measurement. Three recordings were performed for each sample. The settings used for data acquisition and analysis were as follows: camera intensity 10, slider gain 200, acquisition time 60 s, a frame rate of 30 frames/s, temperate 22 °C and detection threshold 3. Data were analyzed using the NanoSight NTA software v3.2.

### Transmission electron microscopy

400 μl of sEV eluate was fixed with 2.5% glutaraldehyde for 24 h at 4 °C and washed 10 min two times with PBS on room temperature. After blocking the light, the samples were fixed with 500 µl of 2% Osmium tetroxide for one hour and dehydrated with 0.5 ml ethanol for 5 min on room temperature. Next, the samples were incubated with 0.5 ml of propylene oxide for 10 min. After discarding the propylene oxide, the samples were incubated in Epon and propylene oxide mixed solution for one hour, and then only in Epon for another hour. The samples were dried and viewed in a transmission electron microscope (JEM-1400).

### Western blot analysis

The sEV eluate and sEV-depleted fraction were concentrated using an Amicon Ultra-4 10 kDa centrifugal filter device (Merck Millipore). The protein concentration was determined via the BCA assay, and 20 μg of protein was resolved on SDS-PAGE, transferred to the PVDF membrane, and blotted with primary antibodies specific for CD63 (10628D, Thermo Fisher Scientific, USA, 1:1000), transferrin (ZRB1225, Sigma-Aldrich, USA, 1:1000), Calnexin (Adi-SPA-860-D, Enzo Life Sciences, Farmingdale, USA, 1:1000) and GM130 (610822, BD Transduction Laboratories, USA, 1:1000). Antibody labeling was detected by incubating blots with horseradish peroxidase-conjugated goat anti-mouse secondary antibody (BR1706516, Bio-rad, USA, 1:4000) and PierceTM ECS Plus Western Blotting Substrate and captured with Amersham Imager 600 (GE Healthcare Life Sciences).

### Small RNA library construction and sequencing

Total RNA was purified from the serum EV isolates using exoRNeasy serum/plasma maxi kit (QIAGEN GmbH, Hilden, Germany) according to manufacturer’s instruction. The serum sample (4 ml) was processed to produce sEV RNA eluate (14 μl). cDNA library was constructed with 10 ng input of RNA, using a SMARTer smRNA-Seq Kit for Illumina following the manufacturer's protocol.

The amplified libraries were purified from 6% Novex TBE-PAGE gels (Thermo Fisher, MA) to excise 138–220 bp (18–100 bp of cDNA plus 120 bp of adaptors) fraction. Resulting library cDNA molecules include sequences required for clustering on an Illumina flow cell. The libraries were gel purified, and validated by checking the size, purity, and concentration on the Agilent Bioanalyzer. The libraries were quantified using qPCR according to the qPCR Quantification Protocol Guide (KAPA Library Quantification kits for Illumina Sequencing platforms) and qualified using the TapeStation D1000 ScreenTape (Agilent Technologies, Waldbronn, Germany). The libraries were pooled in equimolar amounts, and sequenced on an Illumina HiSeq 2500 (Illumina, San Diego, USA) instrument to generate 101 base reads. Image decomposition and quality values calculation were performed using the modules of the Illumina pipeline. All procedures for next-generation sequencing (NGS) analysis were performed by Macrogen (Seoul, Korea).

### Preprocessing and analysis of RNA sequencing data

After sequencing, the raw sequencing reads were filtered based on quality, and the adapter sequences were trimmed off using the Cutadapt program (v2.8)^39^. Sequenced reads are classified as trimmed reads, non-adapter reads, short read (< 17 bp after adapter trimming). If a read matches a least first 5 bp of 3′ adapter sequence, it was regarded as an adapter sequence, and then trimmed from the read. Trimmed reads should be at the minimum of 18 bp to be considered reliable for analysis.

To get a comprehensive profile of small RNA derived from serum EV enriched fractions, we analyzed both short (< 50 bp) and long target (≥ 50 bp). The trimmed reads were used in case of short target, while both trimmed and non-adapter reads were used as processed reads to analyze the long target. Trimmed or non-adapter read with one or more ‘N’ base was regarded as low-quality read and filtered.

The processed reads were gathered forming a unique cluster which contains reads that are 100% match to the sequence identity as well as read length. The cluster is given its temporary cluster ID and the number of reads it holds. To eliminate the effect of large amounts of rRNA, reads were aligned to the rRNA sequence (45S pre-rRNA and mitochondrial rRNA of *Homo sapiens*) and removed.

Final processed reads were sequentially aligned to reference genome (hg19), miRBase (release 21)^40^ and non-coding RNA database (RNAcentral release 10.0^41^) to classify known miRNAs and other types of RNA such as piRNA, tRNA, snoRNA, snRNA, etc. Known and novel miRNAs predicted by miRDeep2 (v2.0.0.8)^42^ and other small RNAs matching RNAcentral were aligned using Bowtie (v1.1.2)^43^ (50 nt or less, piRNA, miRNA, etc.) and Bowtie2 (v2.3.4.1)^44^ (50 nt or greater; tRNA, snoRNA, etc.).

Quantification of known mature miRNA abundance, which was used to analyze differential expression between ALS patients and healthy controls, was performed as follows. First, mature miRNA sequences of human were aligned to precursor miRNA sequences from miRBase. Next, unique clustered reads were aligned to precursor sequence using Bowtie aligner. Afterwards, the read counts of mature miRNAs were extracted from the overlapping regions in the miRDeep2 Quantifier module. Differentially expressed miRNAs were determined through comparison between patient and control groups using statistical methods.

### Droplet digital PCR

We used droplet digital PCR (ddPCR) to validate the differential expression of miRNAs in an independent validation cohort (Table 1). TaqMan miRNA Reverse Transcription kits (Thermo Fisher Scientific, USA) and miRNA-specific primers for miR-23c (463068_mat, Thermo Fisher Scientific), miR-324-3p (000579), miR-192-5p (000491), miR-32-5p (002109), miR-378a-5p (000567), miR-191-5p (002299) were used for reverse transcription. For each sample, we used a fixed volume of RNA eluate (8 μl) purified from 4 ml of serum to minimize the variability in the amount of microRNA across samples.

For ddPCR reaction, cDNA sample, ddPCR supermix for probes, TaqMan miRNA probe and RNase-free water was added in a 20 μl reaction mixture. The mixture was loaded into a droplet generator cartridge (Bio-Rad QX200), and the generated droplets were transferred to a 96-well template for PCR. The thermal cycling conditions were 95 °C for 10 min, 40 cycles of 94 °C for 30 s, 60 °C for 1 min, and a final step at 98 °C for 10 min. A no template control (NTC) was included in every assay. The amplified droplets were analyzed using the Quantasoft software version 1.7.4 (Bio-Rad).

To identify endogenous reference miRNA, we evaluated the stability of expression of miRNAs by using NormFinder software (v0.953) which identified miR-191-5p as the most stable miRNA in our dataset^45^. After normalization using miR-191-5p as an endogenous reference, the level of miRNA was expressed as a relative quantitative value compared to the average value of the healthy control.

### miRNA target genes and functional enrichment analysis

In order to assess the functional relevance of differentially expressed miRNAs, we conducted functional enrichment analysis for the target genes of validated miRNAs. The R-based multiMiR package (version 2.3.0) was used to retrieve the miRNA-target interactions from 11 external databases^46^. Among the 11 external databases, eight contain predicted miRNA-target interactions (DIANA-microT-CDS, ElMMo, MicroCosm, miRanda, miRDB, PicTar, PITA, and TargetScan), and three have experimentally validated miRNA-target interactions (miRecords, miRTarBase, and TarBase). We searched the top 20% among all conserved and non-conserved target sites in each database, and selected target genes identified in at least three databases for the predicted targets and at least one database for the validated targets.

Functional enrichment analysis was performed using g:Profiler through its web interface (https://biit.cs.ut.ee/gprofiler/), and Gene ontology (GO) biological process was used as a gene set database^47^. The enrichment significance was analyzed by using Fisher’s exact test with adjusted p-values (Benjamini–Hochberg FDR < 0.05). Cytoscape and EnrichmentMap were used for visualization of the functional enrichment analysis results^48^.

### Statistical analysis

Data are expressed as the mean ± standard deviation (or median with interquartile range) for continuous variables, and number (percentage) for categorical variables. For differential expression analysis of miRNAs, miRNAs with zero count across more than 50% of all samples per either patient or control group were excluded to avoid bias caused by miRNAs with relatively low expression level. Next, the raw data (i.e., read counts for each miRNA) were normalized using three different methods: Relative Log Expression (RLE) (implemented in the DESeq2 package (v1.20.0))^49^, Trimmed Mean of M-values (TMM) (implemented in the edgeR package (v3.22.0))^50^, and quantile normalization. We added 1 with normalized read count of the filtered miRNAs to facilitate log2 transformation. Statistical hypothesis test for comparison of two groups was conducted using the negative binomial Wald test in DESeq2, the exact test in edgeR, and independent *t* test in quantile normalization. Differentially expressed miRNAs between two groups were determined based on |fold change| ≥ 2 and p value < 0.05. Hierarchical clustering analysis was performed using complete linkage and Euclidean distance as a measure of similarity to display the expression patterns of differentially expressed miRNAs.

Comparison between two groups of miRNA expression measured by ddPCR were performed using Wilcoxon rank sum test. Correlations between miRNAs levels and clinical parameters were assessed using Spearman’s correlation analysis. All data analysis and visualization of differentially expressed genes was conducted using R 3.3.1 (http://www.r-project.org).

## Supplementary Information

Supplementary Information.

## Data Availability

The data that support the findings of this study are available from the corresponding author upon reasonable request.
