# Supplementary material for: Small RNA sequencing of circulating small extracellular vesicles microRNAs in patients with amyotrophic lateral sclerosis
Source: Sci Rep. 2023 Apr 4;13:5528. doi: 10.1038/s41598-023-32717-y (PMC10073149; doi:10.1038/s41598-023-32717-y)
Supplement: Supplementary file 1 — Supplementary Information. [file 41598_2023_32717_MOESM1_ESM.pdf]

Small RNA Sequencing of Circulating Small Extracellular Vesicles MicroRNAs in Patients with Amyotrophic Lateral Sclerosis

Jin-Ah Kim,[1,2†] Canaria Park,[3†] Jung-Joon Sung,[4†] Do-Jin Seo,[5] Seok-Jin Choi,[4] Yoon-Ho Hong[6,7*]

[1] *Department of Translational medicine, Seoul National University College of Medicine, Seoul, Republic of Korea.*

[2] *Genomic Medicine Institute, Medical Research Center, Seoul National University, Seoul, Republic of Korea*

[3] *Department of Neurobiology, Harvard Medical School, Boston, MA, USA.*

[4] *Department of Neurology, Seoul National University Hospital, Seoul, Republic of Korea*

[5] *Department of Neurology, Chung-Ang University College of Medicine, Seoul, Republic of Korea.*

[6] *Department of Neurology, Medical Research Council, Seoul Metropolitan Government-Seoul National University Boramae Medical Center, Seoul, Republic of Korea*

[7] *Neuroscience Research Institute, Seoul National University College of Medicine, Seoul, Republic of Korea*

† Co-first authors

*** Corresponding author:** Yoon-Ho Hong, MD, PhD

Department of Neurology, Medical Research Council, Seoul Metropolitan Government-Seoul National University Boramae Medical Center and Neuroscience Research Institute, Seoul National University College of Medicine

20 Boramaero-5-Gil, Dongjak-Gu, Seoul, 07061, Republic of Korea.

Email: nrhong@gmail.com

Telephone: +82-2-870-2474, Fax: +82-2-831-2826

Supplementary Table 1. Patients' clinical information.

Patient number	Gender	Age	Disease duration (Month)	Onset region	ALSFRS-R	Level of diagnostic certainty
Discovery set						
P1	M	55	11	Bulbar	26	Definite
P2	F	61	10	Limb	35	Probable
P3	F	65	31	Bulbar	31	Definite
P4	F	76	23	Bulbar	26	Probable
P5	M	55	18	Limb	18	Probable
P6	F	37	17	Limb	30	Definite
P7	M	59	10	Limb	37	Definite
P8	M	66	12	Limb	28	Probable
P9	F	50	9	Bulbar	46	Probable-laboratory supported
P10	F	64	10	Bulbar	44	Probable-laboratory supported
P11	F	58	20	Bulbar	31	Definite
P12	M	59	18	Limb	30	Probable-laboratory supported
Validation set						
V1	M	50	5.5	Limb	39	Definite
V2	M	56	5.6	Limb	39	Probable-laboratory supported
V3	F	72	3	Bulbar	40	Probable
V4	M	56	5.4	Bulbar	42	Definite
V5	M	64	5.8	Limb	44	Probable
V6	M	61	6	Limb	45	Definite
V7	M	56	5	Bulbar	45	Probable
V8	M	55	5	Bulbar	29	Definite
V9	M	50	10	Limb	19	Definite
V10	M	56	9	Limb	38	Definite
V11	F	66	5	Limb	43	Probable-laboratory supported
V12	F	60	7	Limb	36	Definite
V13	F	81	5.3	Limb	33	Probable
V14	F	67	5.8	Bulbar	45	Definite
V15	M	56	5.5	Bulbar	41	Definite
V16	M	55	7	Bulbar	34	Definite
V17	F	65	8	Bulbar	23	Definite
V18	F	66	5	Limb	36	Definite

M, male; F, female; ALSFRS-R, ALS functional rating scale-revised

Supplementary Figure 1. Uncropped images of Western blots. Uncropped Western blot images of transferrin (77kDa) (A), CD63 (37-50kDa) (B), Calnexin (98kDa) (C), and GM130 (130kDa) (D)

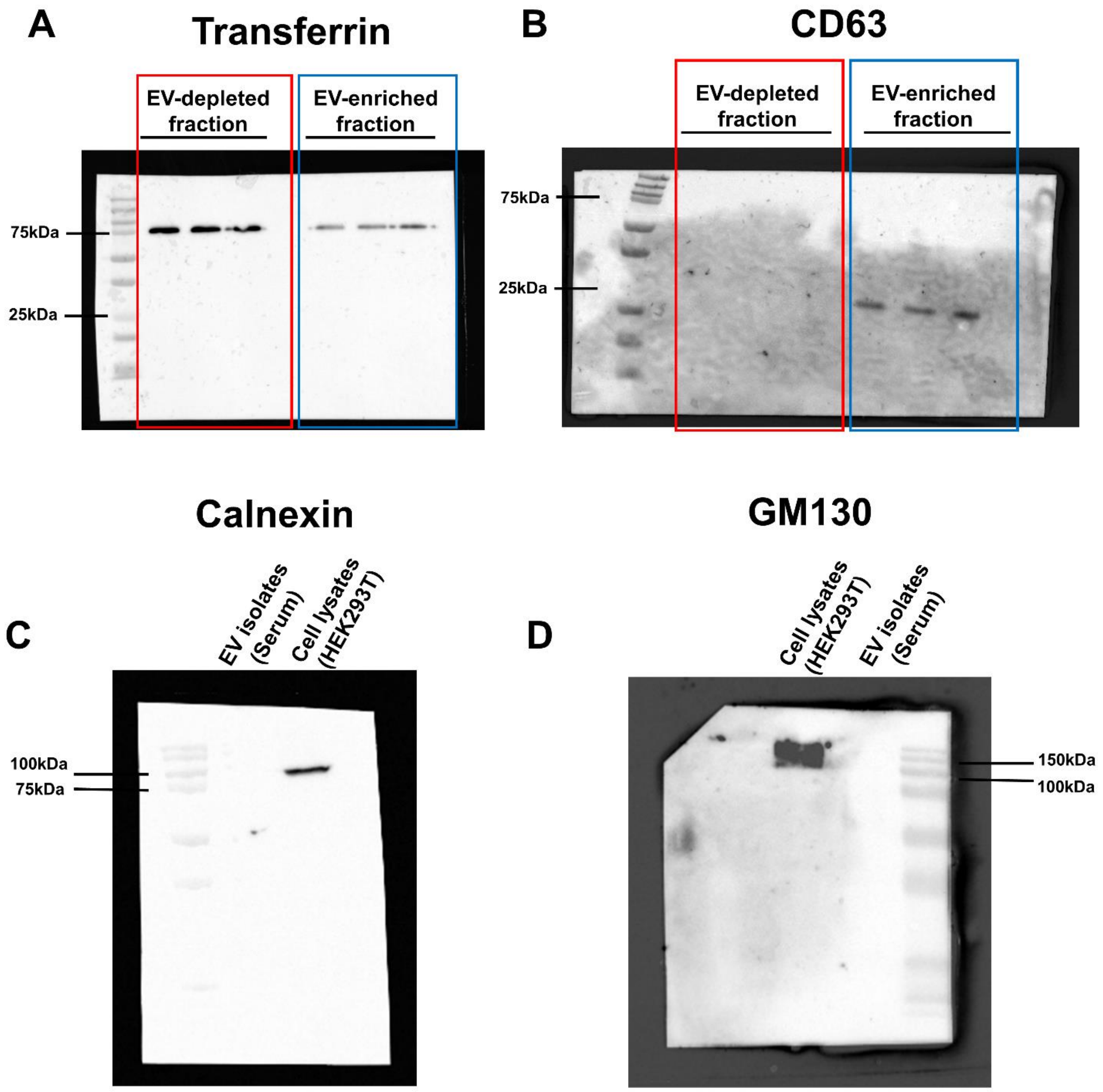

Supplementary Table 2. Summary of read processing. Numbers represent raw counts of the corresponding reads. The reads start at the first base after the 5' sequencing adapter and typically end after 51 bp. As mature miRNAs are normally up to 25 bp in length, the reads will contain part of 3' adapter sequence that has to be removed. If a read matches at least first 5 bp of 3' adapter sequence, it is regarded as an adapter sequence, and then trimmed from the read. Trimmed reads should be at the minimum of 18 bp in order to be considered reliable for analysis. In addition, trimmed or non-adapter read with 'N' base was regarded as low quality read and filtered. Sequenced reads are classified as trimmed reads, non-adapter reads, short reads and low-quality reads according to the following definition: Trimmed Read, Read that is removed adapter sequences; Non-adapter Read, Read that has not adapter sequences; Short Read: Read with below 17 bp in read length after adapter trimming, Low-quality Read: Read with one or more 'N' base in trimmed or non-adapter read. Both trimmed and non-adapter reads were analyzed to see comprehensive small RNA profiles.

Sample	Total Read	Trimmed Read	Non-adapter Read	Short Read	Low-quality Read
C1	34,054,152	16,302,132 (47.87%)	348,469 (1.02%)	17,393,694 (51.08%)	9,857 (0.03%)
C2	29,153,184	9,669,621 (33.17%)	82,691 (0.28%)	19,395,904 (66.53%)	4,968 (0.02%)
C3	43,875,832	16,116,888 (36.73%)	1,744,532 (3.98%)	26,003,705 (59.27%)	10,707 (0.02%)
C4	23,895,479	11,394,718 (47.69%)	171,416 (0.72%)	12,303,429 (51.49%)	25,916 (0.11%)
C5	19,666,679	10,730,775 (54.56%)	392,092 (1.99%)	8,531,159 (43.38%)	12,653 (0.06%)
C6	20,420,623	8,591,291 (42.07%)	185,783 (0.91%)	11,635,318 (56.98%)	8,231 (0.04%)
C7	23,682,022	9,291,036 (39.23%)	151,221 (0.64%)	14,222,829 (60.06%)	16,936 (0.07%)
C8	24,178,483	11,319,389 (46.82%)	219,435 (0.91%)	12,613,443 (52.17%)	26,216 (0.11%)
C9	24,183,218	13,174,220 (54.48%)	556,219 (2.3%)	10,365,374 (42.86%)	87,405 (0.36%)
C10	26,210,781	9,255,895 (35.31%)	217,576 (0.83%)	16,710,072 (63.75%)	27,238 (0.1%)
C11	28,984,881	14,790,149 (51.03%)	463,393 (1.6%)	13,684,704 (47.21%)	46,635 (0.16%)
C12	24,732,679	5,007,423 (20.25%)	115,179 (0.47%)	19,598,446 (79.24%)	11,631 (0.05%)
P1	14,886,671	4,157,080 (27.92%)	15,152 (0.1%)	10,712,328 (71.96%)	2,111 (0.01%)

P2	30,338,701	17,683,705 (58.29%)	619,382 (2.04%)	12,024,624 (39.63%)	10,990 (0.04%)
P3	33,815,917	17,647,256 (52.19%)	188,727 (0.56%)	15,970,849 (47.23%)	9,085 (0.03%)
P4	32,055,313	16,407,543 (51.19%)	810,531 (2.53%)	14,827,373 (46.26%)	9,866 (0.03%)
P5	23,990,215	5,662,082 (23.6%)	105,844 (0.44%)	18,208,857 (75.9%)	13,432 (0.06%)
P6	23,555,073	8,925,460 (37.89%)	59,118 (0.25%)	14,565,980 (61.84%)	4,515 (0.02%)
P7	23,287,639	10,831,014 (46.51%)	960,165 (4.12%)	11,482,849 (49.31%)	13,611 (0.06%)
P8	35,037,627	15,577,105 (44.46%)	1,843,470 (5.26%)	17,588,706 (50.2%)	28,346 (0.08%)
P9	28,371,671	14,268,622 (50.29%)	262,260 (0.92%)	13,805,103 (48.66%)	35,686 (0.13%)
P10	30,844,432	16,904,759 (54.81%)	417,731 (1.35%)	13,458,704 (43.63%)	63,238 (0.21%)
P11	30,187,225	18,877,929 (62.54%)	343,758 (1.14%)	10,939,214 (36.24%)	26,324 (0.09%)
P12	28,114,739	12,325,273 (43.84%)	224,844 (0.8%)	15,533,887 (55.25%)	30,735 (0.11%)

Supplementary Figure 2. Small RNA composition. The distributions of small RNAs are shown as boxplots representing the proportions of each biotype among the final processed reads mapped to the category of small RNA (excluding rRNA). The relatively low proportion of known miRNA (1.52% of the processed reads mapped to the category of small RNA, excluding rRNA) may be explained by size selection and gel extraction of small RNA libraries prior to sequencing. In order to investigate the composition of various small RNA biotypes, we excised a band corresponding to 138-220 bp (18 to 100bp of cDNA plus 120 bp of adaptors). This would have decreased the proportion of small RNAs such as miRNA and piRNA (26-31 bp), while increasing the proportion of larger small RNAs such as tRNA (typically 76-90 bp in length).

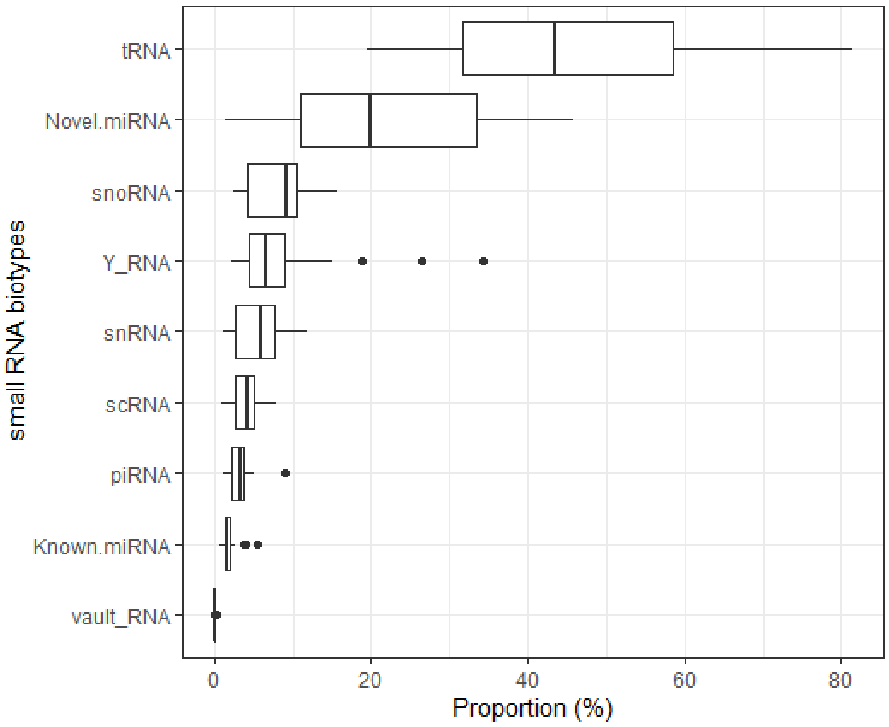

Supplementary Figure 3. Receiver operating characteristic (ROC) curves for the diagnosis of ALS based on the miRNA levels of miR-192-5p, miR-23c and both (fitted by binary logistic regression) measured by small RNA-seq (A, B, C) and ddPCR (D). The miRNA levels were normalized by different algorithms (A: DESeq2, B: EdgeR, C: Quantile normalization), and by an endogenous reference miRNA in ddPCR (miR-191). Diagnostic metrics were summarized in a table below.

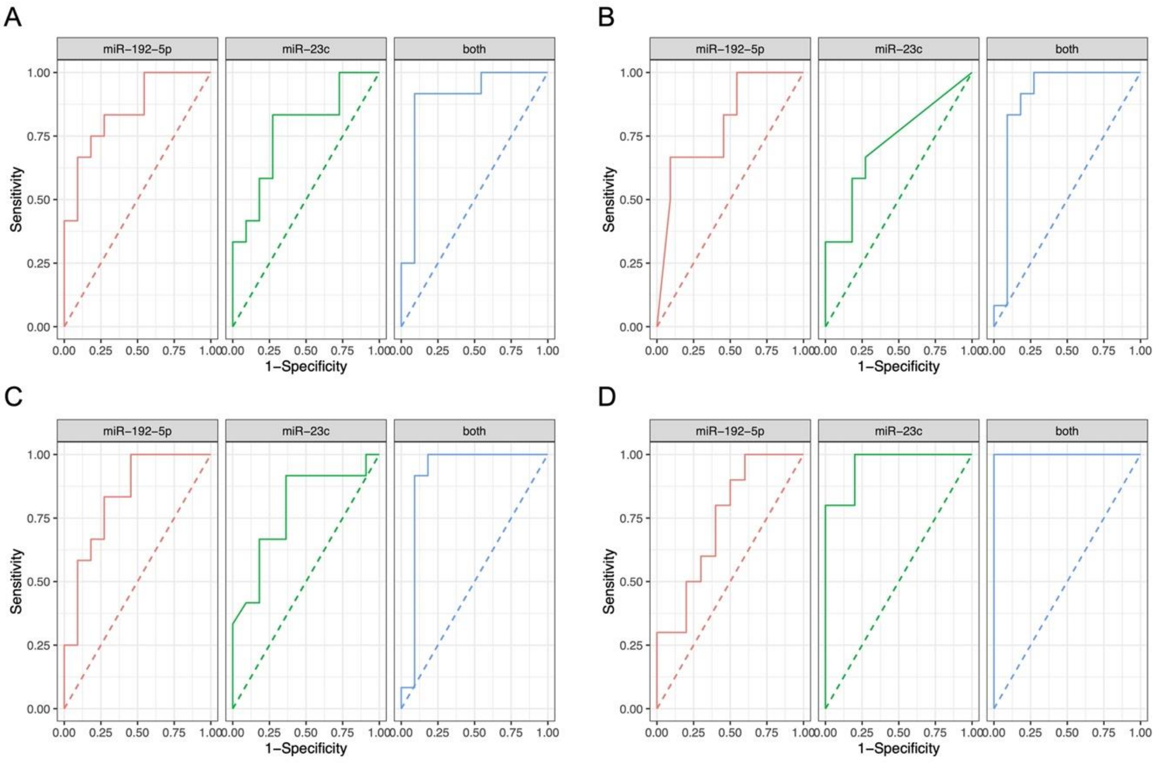

Supplementary Table 3. The measures of sensitivity, specificity, positive predictive value, negative predictive value, and area under the curve in the ROC curves of Supplementary Figure 3.

	Sensitivity	Specificity	PPV	NPV	AUC (95% CI)
miR-192-5p					
DEseq2	0.667	0.909	0.889	0.714	0.848 (0.69-1)
EdgeR	0.667	0.909	0.889	0.714	0.795 (0.607-0.984)
Quantile	0.833	0.727	0.769	0.800	0.833 (0.662-1)
ddPCR	1.00	0.400	0.625	1.00	0.74 (0.515-0.965)
miR-23C					
DEseq2	0.833	0.727	0.769	0.800	0.773 (0.573-0.972)
EdgeR	0.583	0.818	0.778	0.643	0.72 (0.521-0.918)
Quantile	0.917	0.636	0.733	0.875	0.784 (0.586-0.982)
ddPCR	1.00	0.800	0.833	1.00	0.96 (0.849-1)
Both					
DEseq2	0.917	0.909	0.917	0.909	0.894 (0.742-1)
EdgeR	0.833	0.909	0.909	0.833	0.894 (0.728-1)
Quantile	0.917	0.909	0.917	0.909	0.909 (0.745-1)
ddPCR	1.00	1.00	1.00	1.00	1

PPV, positive predictive value; NPV, negative predictive value; AUC, area under the curve

Supplementary Figure 4. A representative figure of multiple correlation plots between the level of miRNA (miR-192-5p and miR-23c) measured by small RNA-seq (quantile normalization) and clinical parameters (onset age, disease duration, and the revised ALSFRS total score). Each panel on the lower triangle is a scatter plot with smooth curve fitted by Loess (locally estimated scatterplot smoothing) for a pair of variables whose identities are given by the corresponding row and column labels. Spearman correlation coefficient for each pair was presented on the corresponding panel on the upper triangle. *p<0.05. All spearman correlation coefficients (p-values within parentheses) were summarized in a table below.

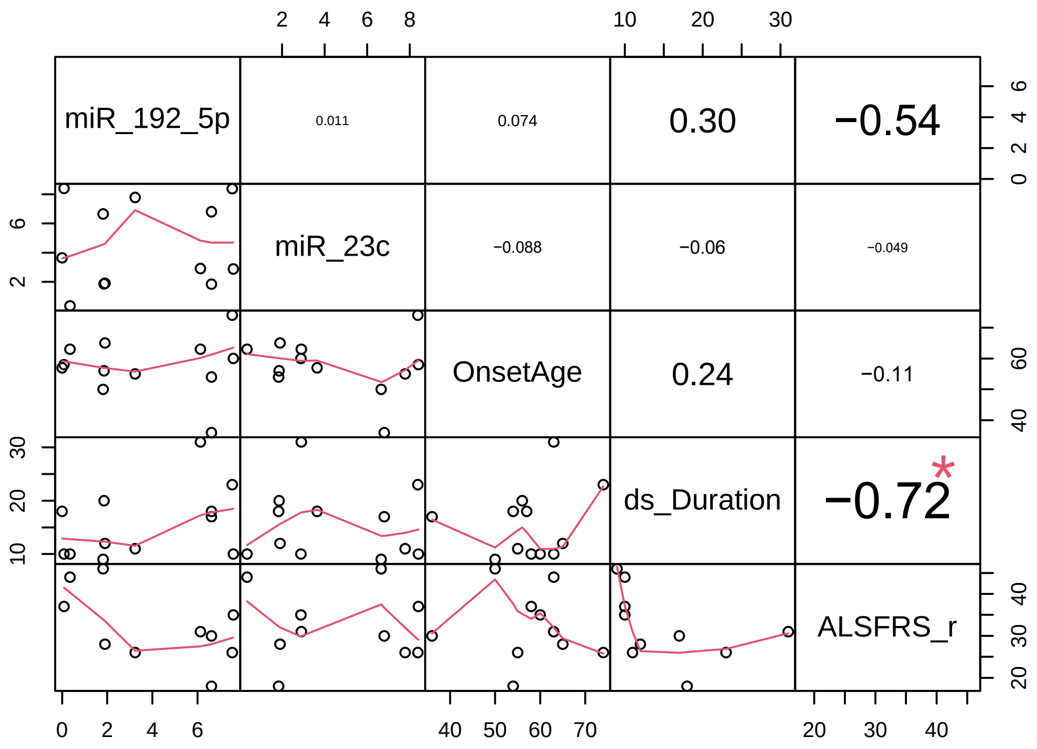

Supplementary Table 4. The correlation coefficients of Spearman correlation analysis in Supplementary Figure 4.

	Onset age (years)	Disease duration (months)	ALSFRS-R total score
miR-192-5p			
DEseq2	0.207	0.620	-0.492
EdgeR	0.110	0.582	-0.458
Quantile	0.055	0.517	-0.543
ddPCR	-0.348	-0.067	-0.067
miR-23C			
DEseq2	-0.018	-0.043	-0.006
EdgeR	-0.092	-0.019	-0.043
Quantile	-0.116	0.031	-0.049
ddPCR	-0.273	-0.104	0.313

Supplementary Table 5. Comparison of the methods and cohort between studies on the circulating EV miRNA in ALS. The discovery and validation cohort are presented separately with the number of patients and controls within parentheses.

Author (year)	Sample	EV isolation	miRNA profiling	Discovery cohort	Validation cohort
Xu et al. (2018)	Serum	Precipitation (ExoQuick)	qRT-PCR	30 (10 vs. 20)	NA
Katsu et al. (2019)	Plasma	Precipitation (polyethylene glycol) & immuno-affinity (L1CAM)	miRNA Oligo Chip	10 (5 vs. 5)	NA
Saucier et al. (2019)	Plasma	Peptide affinity (Venceramin)	NGS (discovery) ddPCR (validation)	26 (14 vs. 12)	15 (12 vs. 3)
Banack et al. (2020)	Plasma	Precipitation (exoQuick) & immuno-affinity (L1CAM)	NGS (discovery) qPCR (validation)	20 (10 vs. 10)	20 (10 vs. 10)
Pregnolato et al. (2021)	Serum	Precipitation (miRCURY)	qRT-PCR	10 (7 vs. 3)	NA
Lo et al. (2021)	Serum	Immuno-affinity (CD63)	NanoString	21 (14 vs. 7)	NA
Present study	Serum	Membrane affinity (exoEasy)	NGS ddPCR	23 (12 vs 11)	33 (18 vs 15)
